# A Feasibility Study on the Recall of Metallophilic Fungi from Fe(III)-Contaminated Soil and Evaluating Their Mycoremediation Capacity: Experimental and Theoretical Study

**DOI:** 10.3390/jof9030382

**Published:** 2023-03-21

**Authors:** Aya I. Tagyan, Manal M. Yasser, Ahmed M. Mousa, Dalal Hussien M. Alkhalifah, Wael N. Hozzein, Marym A. Marzouk

**Affiliations:** 1 Department of Botany and Microbiology, Faculty of Science, Beni-Suef University, Beni-Suef 62511, Egypt; 2 Department of Biology, College of Science, Princess Nourah bint Abdulrahman University, P.O. Box 84428, Riyadh 11671, Saudi Arabia

**Keywords:** Fe(III), metallophilic fungi, *Trichoderma harzianum*, mycoremediation, soil

## Abstract

Mycoremediation is one of the most attractive, eco-friendly, and sustainable methods to mitigate the toxic effects of heavy metals. This study aimed to determine the mycoremediation capacity of metallophilic fungi isolated from heavy-metal-contaminated soil containing a high Fe(III) concentration (118.40 mg/kg). Four common fungal strains were isolated, including *Curvularia lunata*, *Fusarium equiseti*, *Penicillium pinophilum*, and *Trichoderma harzianum*. These fungal strains were exposed to gradually increasing concentrations of Fe(III) of 100, 200, 300, 400, 500, 600, 700, 800, 900, and 1000 mg/L. Sophisticated techniques and tests were employed to investigate the mycoremediation capability, including tolerance index (TI), scanning electron microscopy (SEM), Fourier-transform infrared spectroscopy (FTIR), and adsorption isotherm. Furthermore, the impacts of initial concentration, pH, and temperature on the Fe(III) removal (%) and uptake capacity (mg/g) of the studied samples were investigated. The results were validated by statistical analysis using one-way ANOVA. It was found that the Fe(III) uptake with different ratios triggered alterations in the Fe(III) tolerance (TI) morphological (SEM), chemical (FTIR), and adsorption capacity properties. The highest Fe(III) tolerance for all studied fungal strains was achieved at 100 mg/L. Moreover, the optimum conditions of Fe(III) removal (%) for all studied fungal strains were within pH 7 and 28 °C, with similar performance at the initial Fe(III) concentration ranging from 50–200 mg/L. At the same time, the maximum Fe(III) uptake was achieved at pH 7, 20 °C, and 200 mg/L. Compared to other strains, the Fe(III) tolerance of *T. harzianum* was rise in the Fe(III) concentration. The Fe(III) uptake reaction was corroborated by best fitting with the Langmuir model, achieving optimum adsorption capacities of 61.34, 62.90, 63.30, and 72.46 mg/g for *C.lunata*, *F. equiseti*, *P. pinophilum*, *T. harzianum*, respectively. It can be deduced that the addressed fungi species can be applied in mycoremediation according to the utilized Fe(III) concentrations with more superiority for live *T. harzianum*.

## 1. Introduction

Environmental pollution is one of the most critical problems of the twenty-first century [1]. Soil contamination is one of the different forms of environmental pollution. Soil pollution is caused by the aggregation of high concentrations of toxic compounds, chemicals, salts, radioactive materials, or disease-causing agents that endanger the health of plants and animals [2]. Various chemicals or heavy metals can contaminate soil through agricultural and industrial activities. Due to soil contamination, microorganisms, aquatic life, humans, and animals are at risk. Immense industries are the most common sources of heavy metal contaminations of the soil, including cadmium, chromium, copper, iron, lead, nickel, and zinc [3]. These heavy metals can trigger serious health problems, such as skin irritation, organ damage, nervous system disorders, gastrointestinal issues, and various types of cancer [4].

**Figure 1:**
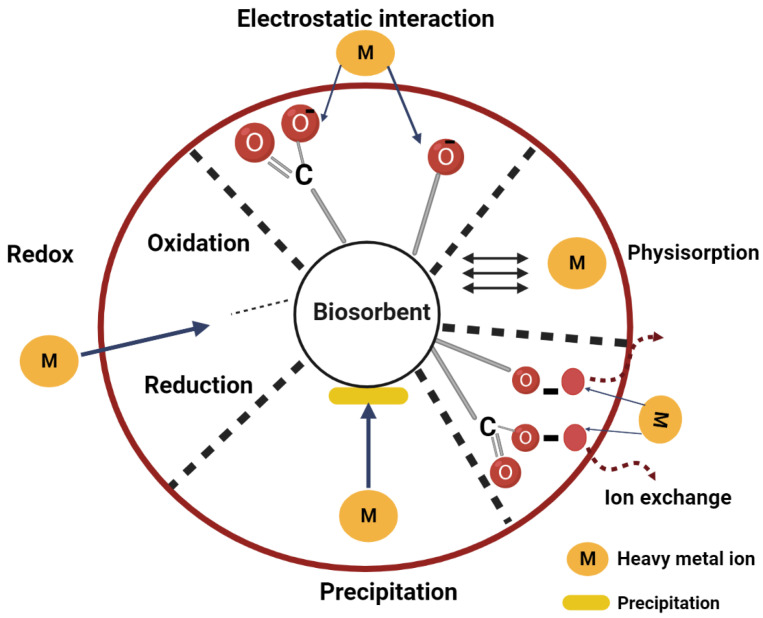
Biosorption mechanisms of heavy metals (M) by fungi.

Iron (Fe(III)), is one of the essential nutrients in plant nutrient cycling and primary mineral weathering [5]; however, it can be toxic in large quantities or under specific formulas. Fe(III) is one of the most common and poisonous heavy metals; it pollutes the soil and causes hazards to biodiversity, agricultural productivity, food safety, and human health when transported through the food chain [6]. However, two processes can describe the removal of such heavy metals from the soil by the live organisms: bioaccumulation and biosorption. Bioaccumulation is an active metabolic uptake process driven by energy from the living organism (e.g., fungi), in which the heavy metals accumulate inside the cell wall (i.e., intracellular binding) [7,8]. On the other hand, biosorption occurs when heavy metals are adsorbed on the cell wall (i.e., extracellular binding). This process can be defined as a fast and passive metabolic process independent of energy in which biological materials (e.g., fungal biomass) act as sorbents that can remove pollutants, such as heavy metals, from wastewater through metabolically mediated or physico–chemical pathways of uptake [8,9]. This process includes mechanisms such as redox, precipitation, electrostatic interaction, physisorption, and ion exchange, as shown in Figure 1.

Mycoremediation is a method of bioremediation in which fungi-based remediation methods are applied to neutralize or remove heavy metals and textile dyes from the environment [10]. Additionally, it is a type of bioleaching remediation that is simple in operation, costless, and biodegradable. Mycoremediation is a novel and promising remediation technology that can be applied to heavy-metal-polluted soils. Therefore, scientists are currently investigating mycoremediation methods that use microbial and associated biota (i.e., fungi) within the ecosystem to biodegrade, collect, and eliminate the contaminants [11]. Hence, the soil (as a part of the ecosystem) is privileged because it contains all main groups of microorganisms, including bacteria and fungi [12]. The microbiota of soil (i.e., bacteria, fungi, and algae) plays an essential role in the degradation and synthesis of organic compounds. Metallophilic fungi are particular fungi that can be isolated from these heavy-metal-polluted soils [13]. These fungi are characterized by their ability to adapt to heavy metals and environmental conditions. The metallophilic fungal tolerance to Fe(III) has been attributed to numerous methods, including metal capture by cell wall components, precipitation by extracellular metabolites, and intracellular complexing by metallothioneins and phytochelatins [14].

Consequently, recent studies have investigated how to isolate species of those fungal strains, which remove heavy metals from different environmental resources such as soil. To the best of our knowledge, bioremediation by these metallophilic fungi has not previously been appointed in iron remediation. Consequently, the current study aims to identify the diversity of these metallophilic fungi present in Fe(III)-contaminated soils and determine their tolerance index at different Fe(III) concentrations. This was conducted through the isolation and characterization of different metallophilic fungal types by employing macroscopic characterization, metal tolerance index (TI), scanning electron microscopy (SEM), and Fourier-transform infrared spectroscopy (FTIR). Meanwhile, Fe(III) adsorption experiments accompanied by their isotherm studies were carried out to evaluate the adsorption capacity of these fungal species.

## 2. Materials and Methods

### 2.1. Soil Collection and Characterization

#### 2.1.1. Soil Collection and Preparation

Soil samples were collected from Bayad Al Arab, an industrial area in East Beni Suef city, Egypt. samples were compiled by plowing the soil layers (0–20 cm) with a sharp-edged plastic spatula from different areas separated by 10–20 m. Then, the soil sample was stored in sterile plastic bags and transferred cautiously to the Microbiology Research Laboratory at the Department of Botany and Microbiology, Beni-Suef University, for further preparation and analysis. Ten soil samples were collected from industrial effluent in plastic bags using a hand trowel at a depth of 20–40 cm. The samples were mixed, and the representative sample was taken and stored for microbial culturing and characterization within 24 h of the collection. The sample was air dried in laboratory conditions for 14 days to obtain a constant mass. The sample was then dried at 75 °C in a hot-air oven and preserved in the desiccator. After that, the sample was ground in a porcelain mortar and sieved through a 2 mm sieve to turn them into fine grains and remove any contaminants of large particles. The sample was then kept in plastic bags for further analysis.

#### 2.1.2. Soil Characterization

pH, electrical conductivity, organic carbon, soil texture, moisture content, and available nutrients (N, P, K, and Fe(III)) were determined. Soil pH was measured in a 1:2.5 aqueous soil extract using a pH meter model (AD8000), whereas electrical conductivity was determined in a 1:5 aqueous soil extract using a conductivity meter (model AD8000). Soil texture was measured by the pipette method according to Olmstead et al. [14]. According to a standard protocol, to determine the available nitrogen content in the soil sample, 5 g of soil was shaken with 50 mL of KCl (2 M) for 30 min, with the moisture content of the soil being determined by Su et al. [15]. The solution was filtered, and the sum of NH_4_^+^ and NO_3_^−^ was measured following the method of Allen et al. [16]. Other available nutrients, including P and K, were determined according to the practices described by Soltanpour and Schwab [17]. Organic carbon was determined based on the method described in [18]. The total Fe(III) concentration was measured by atomic absorption spectrophotometry (Agilent 240 FS AA).

### 2.2. Isolation and Characterization of Metallophilic Fungi

#### 2.2.1. Metallophilic Fungi Isolation

To isolate the fungal strains, 3 g of soil samples was dissolved in 200 mL of sterilized distilled water. The suspension was then serially diluted 10–1000 times at room temperature in a shaking incubator with a shaker at 100 rpm. To inhibit the bacterial growth in the media, the samples were added to 30 mg/L of streptomycin in Potato Dextrose Agar (PDA, Merck KGaA^®^, Darmstadt, Germany). A total of 100 mL of the dilution was added, and the media was poured into Petri dishes (9 cm in diameter). After media solidification, the plates were placed in an incubator at 28 °C in dark conditions and checked daily for 6 days. The growth of different fungal species was observed after 4 days [19]. Each prepared colony was subcultured and preserved on 2% PDA plates at 25 °C ± 2 °C and then stored at 5 °C.

#### 2.2.2. Fungal Characterization

The fungal species used and the different tests conducted in this study were characterized by employing several experiments, including macroscopic characterization, metal tolerance index (TI), scanning electron microscopy (SEM), and Fourier-transform infrared spectroscopy (FTIR).

##### Macroscopic Characterization

For the macroscopic characterization, each isolated strain was separated on fresh PDA plates with 100 mg/L of Fe(III) adjusted to a pH of 5.5 to 5.7 by incorporating FeCl_3_·6H_2_O (Sigma-Aldrich, Munich, Germany).

##### Tolerance Index (TI)

Relating to the metal tolerance characterization, the tolerance index (TI) is defined as the ratio of the treated colony radial growth rate incorporated with Fe(III) to that of control of the same fungal isolate without Fe(III) incorporation. The potential of Fe(III) tolerance of the fungal colonies in the test medium was measured according to Equation (1).

(1) $$ΤI=\frac{\mathrm{Dt}}{\mathrm{Du}}$$

where TI is the tolerance index, Dt is the plate radial diameter (mm) treated with Fe(III), and Du is the untreated plate radial diameter (mm).

The tolerance to Fe(III) of the fungi was evaluated as follows: 0.00–0.39 (very low), 0.49–0.59 (low), 0.60–0.79 (moderate), 0.80–0.99 (high), and ≥1 (very high) [20]. The tolerant strains were cultivated in various concentrations of Fe(III), including 0, 100, 200, 300, 400, 500, 600, 700, 800, 900, and 1000 mg/L of Fe(III). This was conducted through the incorporation of FeCl_3_.6H_2_O (Sigma-Aldrich, Munich, Germany) in PDA media. The experiment was conducted in triplicate for both the control and each metal concentration. After 7 days, fungal discs of 3 mm size were inoculated onto the PDA media at 27 °C, while the media without Fe(III) served as control. After 7 days, the mycelium discs (3 mm diameter) were separately inoculated from the pure cultures, after which the plates were incubated for equal days with monitoring of the radial growth of the mycelium at the same temperature.

##### Colony Morphology

After fungi colony cultivation, as previously mentioned, macroscopic characteristics, colony color, colony texture, medium changes, and pigment formation were described. The colony diameters were also photographed after 7 days at 27 °C. For each colony, the average of two perpendicular diameters was calculated. The names of colors were referenced by Ridgway [21].

##### Scanning Electron Microscopy (SEM)

The dried fungal biomass was evaluated before and after the contact with Fe(III) solutions. After that, the dried fungal biomass was coated with an about 15 nm thickness of gold under vacuum to enhance conductivity, and scanning electron microscopy (SEM, JSM-6510 LA, JEOL, Japan) was employed at 20 kV to determine the morphological modifications that occurred to the fungal mycelia due to Fe(III) addition.

##### Fourier-Transform Infrared Spectroscopy (FTIR)

Based on the absorption bands at definite frequencies, FTIR is an effective technique for identifying the functional groups of specific biopolymers (e.g., proteins, carbohydrates, and cellulose) [22]. Powdered *T. harzianum* was nominated to be examined by FTIR based on the tolerance index value, for which it had the highest values. It is known that all addressed fungi have the same functional groups; thus, the powder from stalks of champignon was evaluated before contact with the Fe(III) solutions. In this study, operating the usual spectral resolution at the usual spectral resolution of 4 cm^−1^, the FTIR technique (VERTEX 70, Bruker, Germany) was used to determine the changes in the functional groups of isolated fungi in the range from 4000 to 400 cm^−1^, due to the addition of different Fe(III) concentrations.

### 2.3. Experimental Work of Adsorption

#### 2.3.1. Adsorption Experiments

Different concentrations of FeCl_3_.6H_2_O (50, 100, 150, and 200 mg/L) were added to potato dextrose broth to evaluate the adsorption capacity (also known as biosorption) of the fungus before the specific fungi were inoculated. As for the effect of pH on the fungal uptake capacity, the fungi cultures were inoculated on media with 200 mg/L of FeCl_3_·6H_2_O, which was adjusted with aqueous HCl and aqueous NaOH to obtain pH values ranging from 3 to 8. The effect of temperature was also studied by incubating the cultures in FeCl_3_·6H_2_O with pH 7 and a concentration of 200 mg/L at 20, 28, and 37 °C. Then, the filtrate from each previous experiment was obtained and analyzed by atomic absorption spectrophotometry to determine its Fe(III) concentration. Next, the biomass of the fungi harvested after filtration was washed with distilled water. Afterward, the biomass was dried overnight in a hot air oven at 80 °C and weighed again. The Fe(III) uptake capacity (qe, mg/g) and Fe(III) removal percentage R (%) were measured by Equations (2) and (3), respectively:

(2) $$\mathrm{qe}=\frac{(C_{0}-C_{F})V}{M}$$

(3) $$R\;(\%)=\frac{C_{0}-C_{F}}{C_{0}}\times 100$$

where *C*_0_ represents the initial concentration of Fe(III) (mg/L), *C_F_* indicates the final concentration of Fe(III) (mg/L), V (L) is the aqueous medium volume, and M is the dry weight of fungal biomass (g).

#### 2.3.2. Adsorption Isotherms

The adsorption isotherm is the first experimental test used to determine whether adsorption is feasible and whether additional test work is required. An equilibrium test provides information linking the amount of adsorbate still in the solution to the amount of adsorbate that was desorbed per unit weight. Adsorption data were reported by adsorption isotherms, such as the Langmuir or Freundlich isotherms, for a wide range of Fe(III) ion concentrations (adsorbate) (50–200 g/1000 mL). The Langmuir and Freundlich models are provided in (Table 1). According to the Langmuir model, maximum adsorption occurs when a saturated monolayer of solute molecules covers the adsorption surface, the adsorption energy is constant, and there is no adsorbate molecule migration in the surface plane. The Langmuir isotherm model expounds on the monolayer adsorption process at the homogeneous interface [23]. The Freundlich isotherm model illustrates the multilayer adsorption at the heterogeneous interface [24]. The Freundlich model is essentially empirical. Therefore, the model is a helpful tool for describing data.

### 2.4. Statistical Analysis

The data presented are the average results of three separate experiments, each of which was performed in triplicate. Version 8.0 of GraphPad Prism was used for statistical analysis and graphic representation. The Tukey test calculated one-way ANOVA and the mean differences between the samples. *p* values that were less than 0.05 were regarded as significant.

## 3. Results and Discussion

### 3.1. Soil Characterization

The mean values of soil analyses are determined in association with standard deviations (SD) and listed in Table 2. The soil pH range was 7.38, indicating that the soil is almost neutral with a slight tendency toward alkalinity. As for the electrical conductivity (EC), the soil sample has a high EC value (25.30 mS/m). This can be attributed to the high concentration of salts in the soil and heavy metal accumulation [27]. The organic matter content of the contaminated soil sample is 0.33%, with a sandy loam texture, and the moisture content is 2%. This result can be attributed to the low concentration of clay in the soil sample. Available nutrient concentrations in soil are represented by nitrogen (N), phosphorus (P), and potassium (K) with mean values of 16, 8, and 10.35 mg/kg, respectively, with SD values ranging from 0.05–0.40. Fe(III) was detected in a high concentration (118.40 mg/kg), above the ISI permissible limits for industrial effluents. This can result from long-term irrigation with industrial effluents [28]. This high concentration of Fe(III) can be a problematic issue in the efficiency of the nutrient cycle due to the reduction in the waste breakdown and nitrogen fixation [29].

### 3.2. Isolation and Characterization of Metallophilic Fungi

#### 3.2.1. Macroscopic Characterization

From the contaminated soil sample, varieties of metallophilic fungi, such as *Curvularia lunata, Fusarium equiseti, Penicillium pinophilum,* and *Trichoderma harzianum*, were isolated. These species have a high resistance to heavy metals [30]. Table 3 illustrates the macroscopic characteristics of these isolated fungi.

#### 3.2.2. Tolerance Index (TI)

**Figure 2:**
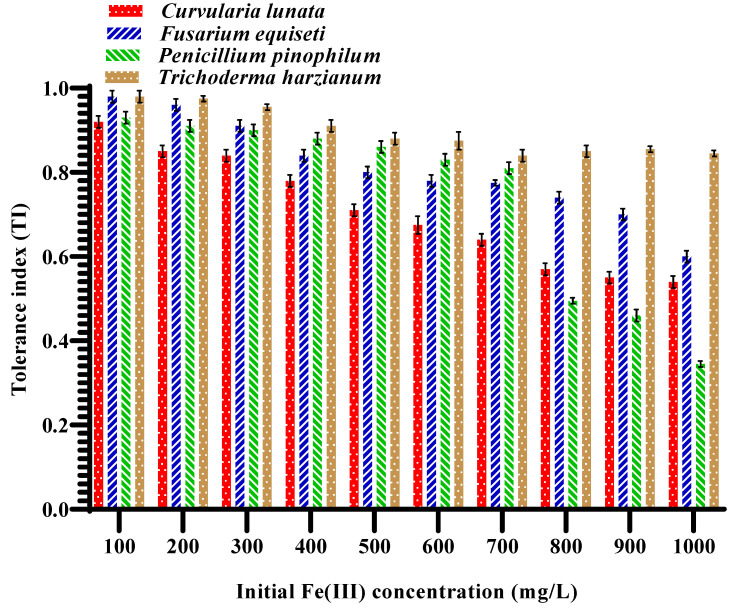
Mean values of tolerance index (%) associated with standard errors (i.e., error bars) of the four isolated fungi at different concentrations of Fe(III) ranging from 100–1000 mg/L.

The TI values of all isolated fungi are < 1 with different ratios ranging from 0.35 to 0.98, which depend on the difference in the tolerance behavior of each fungus, which can be observed from the presence of filamentous fungi in contaminated sites (Figure 2). These differences can be attributed to the different tolerance mechanisms of these microorganisms to metal contaminants [31]. Figure 2 shows the highest Fe(III) tolerance values in all experienced Fe(III) concentrations (100–1000 mg/L). Unlike *P. pinophilum*, *T. harzianum* species can resist and detoxify Fe(III) pollutants even with higher concentrations (i.e., 800–1000 mg/L). More specifically, the change in the Fe(III) tolerance of *T. harzianum* is limited at all Fe(III) concentrations, even at the highest values.

#### 3.2.3. Colony Morphology

**Figure 3:**
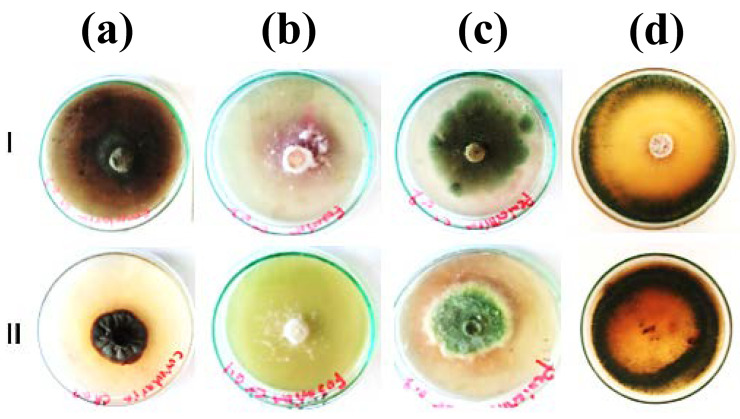
Radial growth diameters of colonies: (**a**) *Curvularia lunata*, (**b**) *Fusarium equiseti*, (**c**) *Penicillium pinophilum,* (**d**) *Trichoderma harzianum*, (**I**) PDA media with no metal, and (**II**) PDA media supplemented with 100 mg/L of Fe(III).

The results of the radial growth diameters and morphological characteristics are illustrated in Figure 3. The *C. lunata* colony is wrinkled in the case of the Fe(III)-supplemented media (Figure 3a). The pink color mycelia of *F. equiseti* changed to white, and the growth of the fungus was reduced in the Fe(III)-supplemented media (Figure 3b). A possible explanation for these changes in the colony color and growth rate can be due to the detoxification mechanisms of heavy metals [32]. *P. pinophilum* is accompanied by a white color around the colony in the media supplemented with Fe(III) (Figure 3c). This can be due to an adaptation period during which Penicillium cells produce enzymes required for Fe(III) uptake [33]. Green colonies of *T. harzianum* mycelia turned dark green with orange pigmentation along the edges (Figure 3d). This can be attributed to the pigment production and metal ion chelation on the fungal cell wall [34]. Compared to other fungal strains, *T. harzianum* has the highest radial growth, signifying the highest Fe(III) tolerance.

#### 3.2.4. SEM Analysis

**Figure 4:**
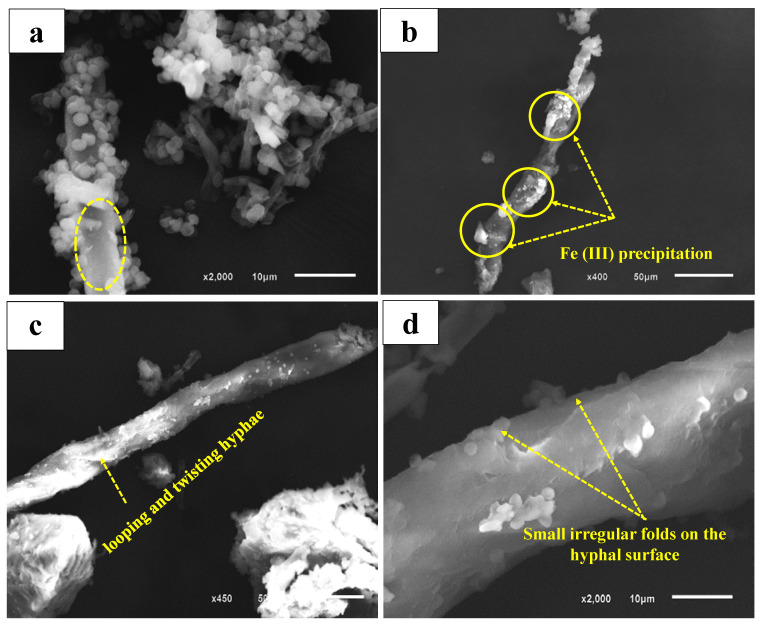
SEM photomicrographs showing the effect of Fe(III) on fungal mycelia. (**a**) control sample without Fe(III) uptake, (**b**,**c**) 50 μm, and (**d**) effect of Fe(III) uptake on fungal mycelia.

SEM images (Figure 4) illustrate alterations in the morphological features of isolated fungi after the uptake of Fe(III). Figure 4a exhibits a regular and smooth surface of the mycelia of *F. equiseti* in the normal status without Fe(III) uptake. Fe(III) precipitate is observed on the mycelial surface of *F. equiseti* due to the Fe(III) bioaccumulation, reflecting the rough surface morphology of these mycelia (Figure 4b). This can be attributed to effective potential biosorption sites on the mycelia surfaces [35]. Furthermore, the bioaccumulation of Fe(III) produces changes in the morphological features of fungi represented in mycelial looping and twisting of *P. pinophilum* (Figure 4c) and small irregular folds on the *T. harzianum* hyphae (Figure 4d). All previous alterations are destructive changes with different grades that occur to the mycelia due to the penetration of Fe(III) into the cell wall. These findings are consistent with those of previous studies [36].

#### 3.2.5. FTIR Analysis

**Figure 5:**
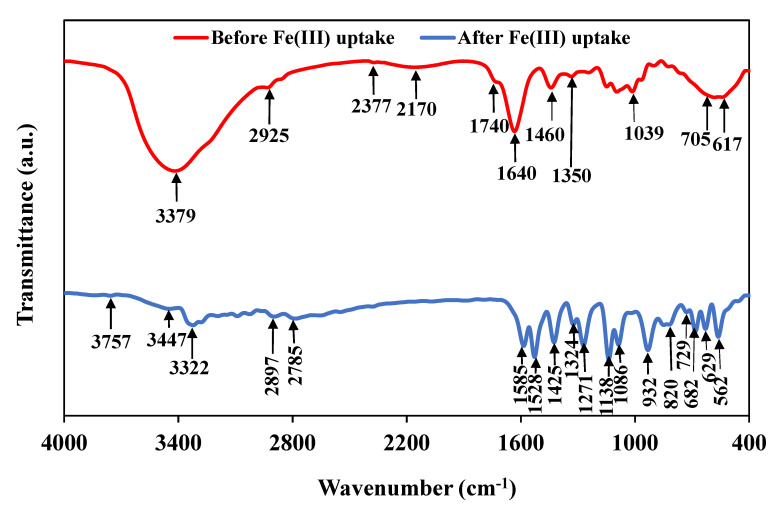
FTIR spectra of *T. harzianum* before and after Fe(III) uptake.

FTIR spectra of the *T. harzianum* before and after Fe(III) uptake are shown in Figure 5. Generally, negatively charged functional groups, such as hydroxyl, amino, carboxyl, phosphate, nitro, and halide groups, provide the electrostatic force required for binding positively charged Fe(III) to the cell surface [37]. The two spectra have some similarities in their profile. The broad band at 3322–3379 cm^−1^ is related to the −NH_2_ and O−H present in carbohydrates or proteins, −NH stretching of amine (protein), and the acetamido group (chitin) [38]. Comparing the two FTIR spectra, a pronounced depletion in this band indicates that the O−H and N−H groups are bound with Fe (III). The presence of a peak at 2925 cm^−1^ is related to the C−H vibration group [39], which has lower intensity after Fe(III) uptake, indicating the role of C−H. The band at 2170 cm^−1^ is reduced in the case of Fe(III) uptake, signifying that C−H, C=O, and C=N contribute to Fe(III) adsorption. Loss of the band in the Fe(III)-laden biomass compared to the band at 1740 cm^−1^ in the case of raw biomass implies the role of C=O stretching vibration in carboxylates of acidic polysaccharides in the metal uptake [39]. In the raw biomass, the band at 1640 cm^−1^ is extinct in the case of F(III)-loaded biomass. This signifies that N−acetyl glucosamine or O−H stretching vibration (i.e., polymer of the protein–peptide bond) has significant support in Fe(III) treatment [40]. The disappearance of peaks at 1460 cm^−1^ at the Fe(III)-loaded sample indicates the role of methylene/alcohol groups (C−H, O−H bending) in the Fe(III) uptake [41]. The peaks at 1039 cm^−1^ belonged to the C–C, C=C, C–O–C, and C–O–P groups of polysaccharides [41]. This peak disappears in the Fe(III)-loaded sample, indicating the potential of these groups in Fe(III) uptake. The bands less than 1000 cm^−1^ concern the fingerprint zone of phosphate and sulfur groups. The decrease in the peak intensity at 617 and 705 cm^−1^ in the metal-loaded strain is attributed to the C−H effect [41]. Moreover, there are noticeable changes in these bands when Fe(III) uptake occurs, signifying that these bands are affected by Fe(III) adsorption. The previous Fe(III) uptake represents a type of biosorption process.

### 3.3. Adsorption Studies

#### 3.3.1. Effect of Initial Fe(III) Concentration

**Figure 6:**
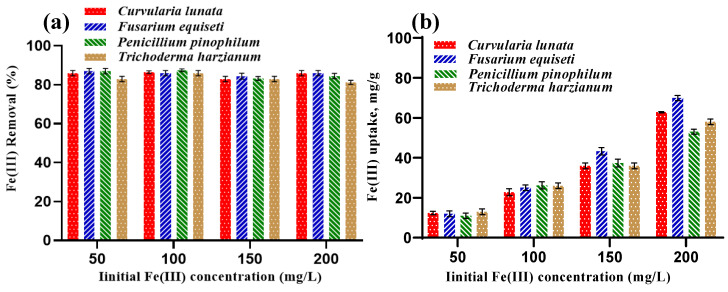
Effect of initial Fe(III) concentrations on (**a**) Fe(III) removal (%) and (**b**) Fe(III) uptake (mg/g).

The effect of initial Fe(III) concentration on adsorption is depicted in Figure 6. The results show that Fe(III) removal (%) increases from 81.25% to 87.50% with the increase in Fe(III) concentration from 50 to 200 mg/L (Figure 6a). Therefore, the Fe(III) removal (%) has an almost similar behavior with minor differences at all initial Fe(III) concentrations, including 50, 100, 150, and 200 mg/L. A possible explanation for this increase can be explained in that this initial Fe(III) concentration can provide the necessary driving force to overcome resistance to Fe(III) ions transfer between aqueous and solid phases [42]. When the initial concentration is low, there are plenty of available adsorption sites, and adsorption can reach equilibrium quickly [43]. In the same Fe(III) concentration range, the findings show that the Fe(III) uptake capacity increased gradually from 10.97 to 62.8 mg/g from 50–200 mg/L, with the highest value at 200 mg/L (Figure 6b). These outcomes can be attributed to the fact that high Fe(III) concentrations can enhance the interaction of Fe(III) ions with biomass, resulting in a higher Fe(III) uptake capacity [44].

#### 3.3.2. Effect of pH

**Figure 7:**
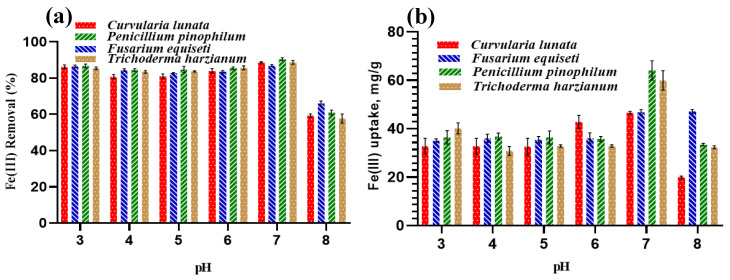
Effect of pH on (**a**) Fe(III) removal (%) and (**b**) Fe(III) uptake.

Fe(III) is a cationic metal with a surface charge of positively charged ions. The Coulombic interaction between positively charged Fe(III) and negatively charged adsorbents can explain why it is more readily adsorbed in acidic environments [45]. As shown in Figure 7a, when the initial pH of the solution lies in the range of 3–7, all isolated fungi perform well in Fe(III) removal (%). More specifically, Fe(III) removal (%) of all fungal strains exceeded 80%. On the other hand, the Fe(III) removal (%) decreases for all isolated fungi at pH 8. As the pH rises, the fungal uptake capacity increases until reaching pH 8 (Figure 7b). Notably, the Fe(III) removal (%) and uptake capacity (mg/g) have almost the highest values for all fungal strains at pH values of 3 and 7, with more superiority for the second (i.e., pH 7). Although it is supposed that pH 7 is similar to pH 8 in terms of having the same deleterious impact on the Fe(III) removal (%) and uptake capacity (mg/g), this does not occur. The unfamiliar behavior at pH 7 can be confirmed by the values of Fe(III) uptake by *P. pinophilum,* and *T. harzianum,* which are equal to 64.28 and 60 mg/g, respectively. This can be attributed to the fact that the fungal strains turn the pH of the medium into acidic status as a result of their secretion of organic acids, including citric, gluconic, malic, itaconic, lactic, and fumaric acids [46]. This was validated by measuring the pH of the medium solution, which is assumed to be 7. However, it was measured and found to be closer to 3 after adsorption. These excreted organic acids not only have an effect on the pH, but also a positive effect on the Fe(III) removal (%) and uptake capacity (mg/g). Hence, there is a difference in the behavior of Fe(III) removal (%) and uptake capacity (mg/g) between the two values of pH (i.e., 3 and 7). This low pH triggers the protonation of binding sites on the microbial surface, thus imparting a negative charge and promoting Fe(III) binding on the microbial surface. On the other hand, the reduced uptake of F(III) ions at pH 8 might be attributed to the accumulation of metal ions inside the cell walls or cells by a combined sorption microprecipitation mechanism [47].

#### 3.3.3. Effect of Temperature

**Figure 8:**
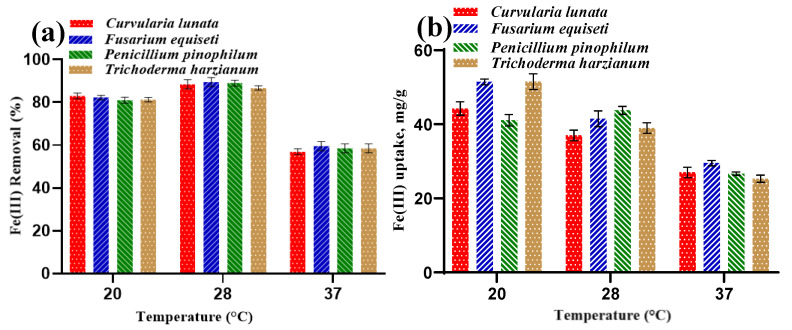
Effect of temperature on (**a**) Fe(III) removal (%) and (**b**) Fe(III) uptake.

It was found that the temperature significantly impacts the biosorption process. At the temperature values of 20 and 28 °C, the Fe(III) removal (%) of fungal strains increases during the temperature increase, reaching a maximum value at 28 °C (Figure 8a). At 37 °C, the Fe(III) removal (%) has the lowest value. At 28 °C, *F. equiseti* has the highest Fe(III) removal (89.5%). Regarding the Fe(III) uptake (mg/g), it decreases gradually from 20 to 37 °C, respectively, and is the highest at 20 °C. More specifically, *T. harzianum* has a maximum Fe(III) uptake of approximately 51.5 mg/g at 20 °C. The findings prove that various fungi have a wide range of temperature adaptability. However, the effect of extremely low- (i.e., under 20 °C) and high-temperature ranges (i.e., above 37 °C) was not investigated for the fungal growth. This can be attributed to the detrimental effect of these temperature ranges on microbial metabolic rates, metal reductase synthesis, and other active materials in the fungal cells [48]. These findings are consistent with previously reported results [35].

### 3.4. Adsorption Isotherm of Fe(III)

**Figure 9:**
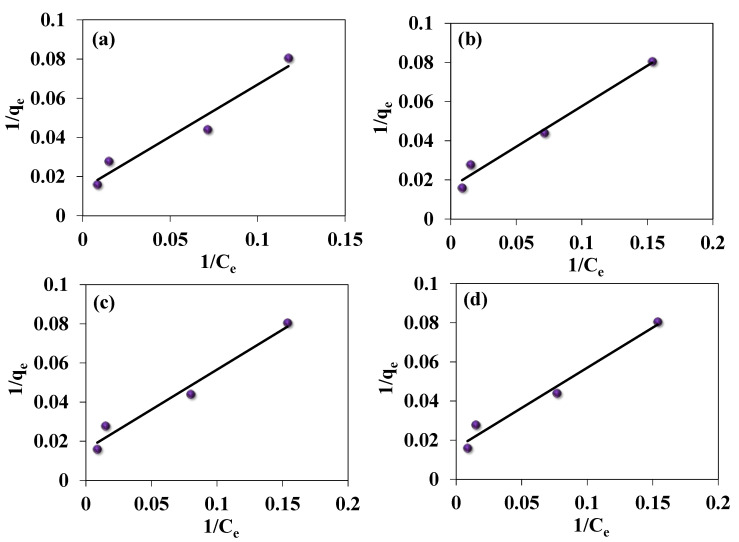
Langmuir isotherm model for Fe(III) uptake by (**a**) *C. lunata,* (**b**) *F. equiseti,* (**c**) *P.pinophilum,* and (**d**) *T. harzianum*.

**Figure 10:**
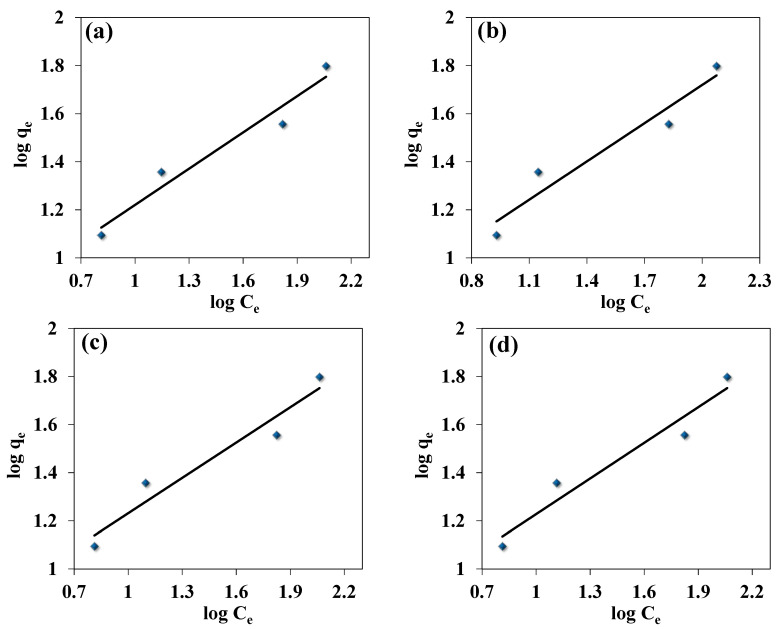
Freundlich isotherm model for Fe(III) uptake by (**a**) *C. lunata*, (**b**) *F. equiseti*, (**c**) *P. pinophilum*, and (**d**) *T. harzianum*.

Figure 9 and Figure 10 show the Freundlich and Langmuir adsorption models applied for the adsorption of Fe(III) ions, respectively. A plot between 1/qe and 1/Ce yields a linear form of Langmuir isotherm. Similarly, the graph plotted between log qe and log Ce yields a linear form for the Freundlich isotherm. Mathematical description and isotherm constants were determined to compare the adsorption capacities of Fe(III) ions for the addressed species. The results have a high value of the correlation coefficients (R^2^) ranging from 0.93–0.98, signifying the adsorption results are the best fit in both the Langmuir model and Freundlich model, with more superiority for the first (Table 4). The high values of Langmuir constants, including q_max_ and K_L_, range from 61.34–72.46 (mg/g) and 0.0390–0.0260 (L/mg), respectively. While the Freundlich constants, including K_F_ and n, range between 4.20–5.60 mg/g and 1.80–2.20 L/mg, respectively. Unlike *C. lunata*, the highest values of Langmuir constants, q_max_ (72.46 mg/g) and K_L_ (0.0260 L/mg), and Freundlich constants, K_F_ (5.60 mg/g and n (2.20 L/mg), are recorded for *T. harzianum*, indicating the highest adsorption capacity and intensity, respectively. This is validated by the highest R^2^ values of 0.94 and 0.95 for Langmuir and Freundlich, respectively (Table 4). Generally, the low absorption efficiency, as observed from the Fe(III) removal (%) and uptake (mg/g), of these live fungal strains is due to their active cellular metabolism, which inhibits the biosorption process.

## 4. Conclusions

Based on the results mentioned above, the following deductions can be addressed as follows:

(1) Four fungal species, including *C.lunata*, *F. equiseti*, *P. pinophilum*, and *T. harzianum*, were successfully isolated from contaminated soil.
(2) The greatest Fe(III) tolerance was achieved for all fungal strains at 100 mg/L. Unlike *P. pinophilum*, the differences in Fe(III) tolerance of *T. harzianum* were limited at all Fe(III) concentrations, even at the highest values.
(3) The morphological characterization by SEM found that there were efficient potential sites of biosorption on the mycelia surfaces responsible for Fe(III) bioaccumulation. Additionally, the Fe(III) bioaccumulation initiated changes in the morphological features of fungi, which can be observed in mycelial looping and twisting and small irregular folds.
(4) Similarly, the Fe(III) removal (%) had the best performance of all experienced initial Fe(III) concentrations, including 50, 100, 150, and 200 mg/L for all studied samples. Meanwhile, the Fe(III) uptake (mg/g) gradually increased with the rising in the initial Fe(III) concentration from 50–200 mg/L to the highest at 200 mg/L.
(5) For all addressed fungal strains, the pH values of 3 and 7 were found to be the optimum conditions with more superiority for the first value for Fe(III) removal (%) and uptake (mg/g). Regarding the temperature, the Fe(III) removal (%) and uptake (mg/g) had the best behavior at 28 and 20 °C, respectively. More specifically, at 28 °C, *F. equiseti* has the highest Fe(III) removal (89.5%), while the Fe(III) uptake (mg/g) decreases gradually from 20 to 37 °C, respectively, and was the highest at 20 °C. More specifically, *T. harzianum* has a maximum Fe(III) uptake of approximately 51.5 mg/g at 20 °C.
(6) Unlike *C. lunata*, the highest values of Langmuir constants, qmax (72.46 mg/g) and K_L_ (0.0260 L/mg), and Freundlich constants, K_F_ (5.60 mg/g and n (2.20 L/mg), m were reported for *T. harzianum*, indicating the highest adsorption capacity and intensity, respectively.

## Figures and Tables

**Table 1:** Adsorption isotherms models for Fe(III) uptake by the isolated fungi.

Isotherm Model	Formula	Parameters	Refs.
Langmuir	$\frac{C_{e}}{q_{e}}=\frac{C_{e}}{q_{max}}+\frac{1}{k_{L}q_{max}}$	C_e_ (mg/L): equilibrium concentration of the resting Fe(III) in the solution q_e_ (mg/g): the removed amount of Fe(III) at equilibrium q_max_ (mg/g): maximum adsorption capacity k_L_ (L/mg): Langmuir constant q_max_ = 1/slope	[25]
Freundlich	$logq_{e}=logk_{f}+\frac{1}{n}logc_{e}$	C_e_ (mg/L): equilibrium concentration of the resting Fe(III) in the solution qe (mg/g): the removed amount of Fe(III) at equilibrium K_F_ (mg/g): Fe(III) adsorption capacity. n: heterogeneity factor k_F_ = 10^intercept^ 1/n = slope	[26]

**Table 2:** Mean values of soil analyses associated with SD.

Soil Properties	Soil Sample
pH	7.380 ± 0.008
Electrical conductivity (mS/m)	25.30 ± 0.50
Organic carbon (%)	0.330 ± 0.012
Clay (%)	12.50 ± 0.40
Sand (%)	43.33± 0.47
Silt (%)	44.16 ± 0.23
Soil texture	Sandy loamy
Moisture content (%)	2.00
Available nutrients (mg/kg)	
N	16.00 ± 0.20
P	8.00 ± 0.40
K	10.35 ± 0.05
Heavy metal (mg/kg)	
Fe(III)	118.40 ± 2.30

**Table 3:** The macroscopic characteristics of isolated fungi.

Isolated Fungi	Colony Features on PDA Media 7-Day-Old
*Curvularia lunata*	Black colonies with brown-black reverse and fluffy texture
*Fusarium equiseti*	White-pinky colonies with white-pale yellow reverse and dense texture (lanose)
*Penicillium pinophilum*	Green colonies with yellow margins and pale orange-light orange reverse and velvety texture
*Trichoderma harzianum*	White mycelium with green colonies with yellowish reverse and cottony texture

**Table 4:** The isotherm parameters of the Fe(III) adsorption by the isolated fungi.

Adsorbent	Initial Fe(III) Concentration (mg/L)	Isotherm Model					
		Langmuir			Freundlich		
		Parameters			Parameters		
		q_max_ (mg/g)	K_L_ (L/mg)	R^2^	K_F_ (mg/g)	N (L/mg)	R^2^
*Curvularia lunata*	50–200	61.34	0.0390	0.98	4.20	1.80	0.93
*Fusarium equiseti*		62.90	0.0380	0.97	4.50	1.88	0.94
*Penicillium pinophilum*		63.30	0.0395	0.97	5.50	1.90	0.94
*Trichoderma harzianum*		72.46	0.0260	0.94	5.60	2.20	0.95

## Data Availability

All data available through the manuscript.
